# Standardising the Lactulose Mannitol Test of Gut Permeability to Minimise Error and Promote Comparability

**DOI:** 10.1371/journal.pone.0099256

**Published:** 2014-06-05

**Authors:** Ivana R. Sequeira, Roger G. Lentle, Marlena C. Kruger, Roger D. Hurst

**Affiliations:** 1 Institute of Food, Nutrition and Human Health, Massey University, Palmerston North, New Zealand; 2 The New Zealand Institute for Plant and Food Research Ltd, Palmerston North, New Zealand; Cincinnati Children’s Hospital Medical Center, University of Cincinnati College of Medicine, United States of America

## Abstract

**Background:**

Lactulose mannitol ratio tests are clinically useful for assessing disorders characterised by changes in gut permeability and for assessing mixing in the intestinal lumen. Variations between currently used test protocols preclude meaningful comparisons between studies. We determined the optimal sampling period and related this to intestinal residence.

**Methods:**

Half-hourly lactulose and mannitol urinary excretions were determined over 6 hours in 40 healthy female volunteers after administration of either 600 mg aspirin or placebo, in randomised order at weekly intervals. Gastric and small intestinal transit times were assessed by the SmartPill in 6 subjects from the same population. Half-hourly percentage recoveries of lactulose and mannitol were grouped on a basis of compartment transit time. The rate of increase or decrease of each sugar within each group was explored by simple linear regression to assess the optimal period of sampling.

**Key Results:**

The between subject standard errors for each half-hourly lactulose and mannitol excretion were lowest, the correlation of the quantity of each sugar excreted with time was optimal and the difference between the two sugars in this temporal relationship maximal during the period from 2½-4 h after ingestion. Half-hourly lactulose excretions were generally increased after dosage with aspirin whilst those of mannitol were unchanged as was the temporal pattern and period of lowest between subject standard error for both sugars.

**Conclusion:**

The results indicate that between subject variation in the percentage excretion of the two sugars would be minimised and the differences in the temporal patterns of excretion would be maximised if the period of collection of urine used in clinical tests of small intestinal permeability were restricted to 2½-4 h post dosage. This period corresponds to a period when the column of digesta column containing the probes is passing from the small to the large intestine.

## Introduction

A number of tests based on the passive absorption of simple sugars have been used to assess gut permeability as an index of recovery from inflammatory bowel disease (IBD) [1], [2] and from autoimmune diseases such as coeliac disease [3], [4] The ratio of the quantities of urinary lactulose and mannitol excreted during a given period has been used most frequently [5], [6], although a number of workers have used rhamnose rather than mannitol [7], [8]. All three probe sugars are passively absorbed from the intestine, not extensively metabolised, and excreted unchanged in urine in proportion to the quantities absorbed [9].

The division of the quantity of lactulose excreted by the quantity of mannitol excreted i.e. the use of ratios, was postulated to compensate for any differences in the surface area of the intestinal mucosa between subjects [10], [11]. However subsequent work has demonstrated a number of factors that may confound the basis of such comparisons as they render the result dependent upon the type of sugar used and the time period over which the cumulative urinary excretion was determined. Firstly, the rate of excretion of the two ‘reference’ sugars that are assumed to reflect surface area i.e. mannitol or rhamnose, is relatively reduced in a number of inflammatory conditions, a phenomenon that has been attributed to villus atrophy [12]. Secondly, the overall rates of excretion of the two reference sugars differ, that of mannitol being significantly higher than that of rhamnose [13]. Thirdly, the rates of excretion of lactulose and mannitol vary with time from dosage due to the differing permeabilities of the small intestine and colon to these sugars [14], [15] These differences cause the ratio of the quantities of the two sugars to vary with the period of time over which their excretion is determined. Thus, the early peak in mannitol excretion causes the LMR to be lower when cumulative excretion is assessed over 2 hours whilst the later peak in lactulose excretion and lower rate of mannitol excretion 4 hours after dosage will cause the LMR to increase when cumulative excretion is assessed over 4 or more hours [13]. These effects may be magnified in the presence of a pro-inflammatory condition [16]–[18] or a pro-inflammatory stimulus such as a single 600 mg dose of aspirin [19], [20] as these will cause the overall excretion of lactulose to increase [4], [21] and that of mannitol to be reduced [13]. These various problems have led researchers to suggest that temporal profiles of absorption of the two sugars should be considered separately as well as in a ratio [22].

In view of these complexities there is a need for the test protocol to be standardized to facilitate meaningful comparisons between populations. Further, to adopt a time at which urine samples should be collected during which the rates of excretion of the probe sugars are broadly similar between subjects so as to the maximise sensitivity of the test.

Hence the purpose of the current work was to assess the temporal profiles of absorption of lactulose and mannitol in a large sample of fit subjects following administration of either placebo or aspirin in a randomized sequence. Thereby to determine the optimal period over which urine samples should be collected, so as to minimise between subject experimental error with due regard to the maximum retention times of the sugar probes in the stomach and in the small intestine as determined with the ‘SmartPill’ [14].

## Materials and Methods

### Use of Aspirin

Aspirin was used as a pro-inflammatory stimulus on the grounds that it induces a reproducible temporary elevation in permeability as determined by lactose mannitol permeability tests [23]–[26]. The rapid increase in permeability is thought to be due to the topical effect of the drug [27] that involves the partitioning of aspirin into the lipid membrane to alter biophysical properties [28].

### Screening and Experimental Protocol

All participants were recruited from a fit, healthy female population of post graduate students in Palmerton North, New Zealand of mixed ethnicity. Female subjects were chosen so as to avoid any gender differences that could influence the recovery of the sugars due to differences in gastric emptying times [29]. The subjects were screened by a health questionnaire and a clinical interview to exclude participants with GI disorders, those taking ongoing prescriptions or over the counter (OTC) medication, prebiotic, probiotic or vitamin supplements and those who had more than a moderate alcohol intake. Similarly, the screening procedure excluded subjects with any history of current urinary tract infections, vaginal discharge, aspirin sensitivity and a history of smoking. Written consent was obtained from all participants before the start of the study as outlined by the Massey University Human Ethics Committee approval procedure.

Although the menstrual cycle has not been shown to have any effect on the recovery of the sugars [30] or on gastric emptying [31], all experimental sessions in both studies were conducted during periods when the participants were not menstruating. This was done to avoid contamination of the urine samples with blood. Participants were instructed to refrain from taking any NSAIDs for at least a week prior to the test, to refrain from consuming alcohol for three days prior to the test, to avoid exercise on the day prior to and on the morning of the test and to fast overnight before attending the test. Each experimental session commenced at 8.00 am when a baseline urine sample was collected from each subject. The order of administration of the treatments (aspirin or placebo) was randomized and double blinded between the two sessions. Each participant received either a placebo drink (100 ml of water) or 600 mg of soluble aspirin (*Dispirin; Reckitt Benkinser Healthcare, UK*) in 100 ml of water at each session. One hour after receiving the treatment, each subject consumed a solution containing 10 g lactulose (*Duphalac, Solvay Pharmaceuticals, NSW, Australia*) and 5 g D-mannitol (*Sigma-Aldrich, St. Louis, MO, USA)* followed immediately by a drink of water. A total of 500 ml of fluid (including the treatment and sugar solution) was therefore consumed by each participant over the first hour. Half-hourly urine collections were commenced after the ingestion of the sugar solution and continued for a period of 6 hours. No food was consumed during the entire urine collection period but a further 200 ml of water was given three hours after the ingestion of the sugar solution. Hence the total amount of fluid administered over the seven hour period was 700 ml. This regime was adopted so as to facilitate half-hourly urine sample collection.

Ethics approval for this part of the study was granted by the Massey University Human Ethics Committee Southern A: 09/79, 11/37. Urinary lactulose and mannitol were quantified with an HPLC system (Shimadzu, Japan) with a refractive index detector using a method described and validated in a previous trial [13], [15].

### Determination of Transit Time of the Sugars Solutions through the Stomach and Small Intestine

Gastric and small intestinal transit time were determined using a wireless pressure and pH sensitive capsule, the SmartPill (SmartPill Corporation, Buffalo, NY) [14] (see below) in six healthy female subjects between 20–40 years of age (mean age: 30 years) who were recruited from the same population and who had been similarly screened. As in the permeability study, each participant attended the laboratory at 8.00 am following an overnight fast. On arrival at the laboratory, each participant consumed a 250 ml drink of water. Fifteen minutes later they were fitted with the SmartPill receiver/data-logger which was attached to a lanyard that maintained it in close proximity to the stomach. They then swallowed the activated and calibrated SmartPill device along with a 250 ml drink containing 10 g lactulose, 5 g mannitol and 5 g glucose. Hence the volume of water consumed was identical to that used in the first study. Each participant was subsequently monitored for seven hours [32]. As in the permeability study, the participants were not allowed to eat or drink during the study period but were given a further 200 ml water mid-way through the session. Ethics approval for this part of the study was obtained from the Massey University Human Ethics Committee Southern A 12/42.

A body of work has been published describing the use of the SmartPill in determination of gastric and small intestinal transit times [33]–[35] to solids and liquids and the device has been approved for the determination of these parameters in a clinical context [14]. The use of the motility capsule is considered a viable alternative to scintigraphy in the assessment of whole gut transit time [36]. The transit times are calculated from changes in the temporal profile of pH and temperature from the data-logger using the Motili*GI* software supplied [14], [32], [33], [36]. Hence gastric residence time was determined as the interval taken from the time of ingestion of the capsule to that of the subsequent rise in pH above that of the asymptotic intra gastric decline on entry into the duodenum [36] (>3pH units). It is noteworthy that such retention time was likely to have been at or above the maximum residence time of the ingested liquid as a body of evidence indicates that particulate solids of greater than 1 mm in linear dimension exited the stomach after concurrently ingested liquid [35] i.e. during phase III of the MMC cycle [37], [38]. The small bowel residence time was taken from the time at which the capsule entered the duodenum to that on entry into the caecum when the pH dropped by at least 1 pH unit [33] below the mean small intestinal pH i.e. pH>4 [32], on entry into the caecum.

### Data Analysis

For the first part of the study, the quantities of lactulose and mannitol in each half-hourly urine sample were determined by multiplication of the relevant concentration (mg.ml^−1^) and sample volume and the result expressed as a percentage recovery of the ingested dose. Data analyses were conducted in the SYSTAT statistical software package version 13 (Systat Software Inc., Chicago, IL) [39].

The mean gastric and small intestinal residence times determined by passage of the SmartPill were used as reference points for the partitioning of the temporal profiles of urinary excretion according to the times of transit of the column of digesta containing the probes into and out of particular segments of intestine.

The temporal patterns of half-hourly excretions were considered as a sequence of separate relationships and the overall changes in the percentage recoveries of the various sugars were evaluated in each, both with and without concurrent dosage with aspirin. Hence the values in the 2½–4 h and the 4½–6 h groups were each regressed against time and the slopes obtained on simple linear regression compared by Students *t* test with respect to group, to sugar and to treatment, with a probability of <0.05 (one tail) being considered as significant.

Mean hourly percentage recoveries for each sugar were derived from the relevant cumulative recoveries over the various time intervals, i.e. 2 h and 4 h. Log converted mean hourly percentage recoveries of each sugar were checked for parametricity by the *Lilliefors* test prior to analysis by doubly repeated measure ANOVA for the effect of treatment and time.

To assess whether any differences in the cumulative recoveries of each sugar could influence the LMR in healthy individuals and in patients with IBDs, we compared the results of our work with those of published studies on subjects with Coeliac or Crohn’s disease [40]–[42] that employed similar protocols and doses of lactulose and mannitol. We determined cumulative recoveries over a 5 h period for each sugar, both after dosage with placebo and after dosage with aspirin, to allow meaningful comparisons with the five hour recoveries reported in these studies. Where possible, the percentage recoveries and associated standard errors were calculated from the reported values in the studies.

Comparisons of the temporal pattern of excretion of mannitol with that of published blood glucose values after standard glucose tolerance test were made by assuming that a blood glucose value in the latter represented the outcome of the instantaneous rate at which glucose was absorbed from the gut vs. that at which it was absorbed by the tissues from the blood. Hence the published temporal sequence of blood glucose values could be converted to quantities of glucose required to maintain the recorded glucose levels above fasting levels over a sequence of half-hour periods by calculating the requisite area under the curve according to the method of Tai *et al* [43].

## Results

None of the forty sedentary, non-smoking, female participants who completed the first part of the study (mean age: 28.2 years) experienced any untoward gastrointestinal effects following consumption of the sugar solutions or aspirin. No subjects complained of thirst during the period of urine collection. The overall mean half-hourly urine volumes were 50.9 (±5.3) ml and 50.9 (±4.6) ml following the consumption of the placebo drink and the aspirin drink respectively. The mean pooled six hourly urine volumes were 878.23 (±63.33) ml and 826.76 (±50.46) ml following the consumption of the placebo drink and the aspirin drink respectively.

None of the six sedentary, non-smoking, female participants who participated in the second part of the study (mean age: 30 years) experienced any untoward effects following dosage with the sugar solutions or the SmartPill and voided the latter within 27–120 hrs.

### Trends in the Percentage Recovery of the Two Probe Sugars

**Figure 1:**
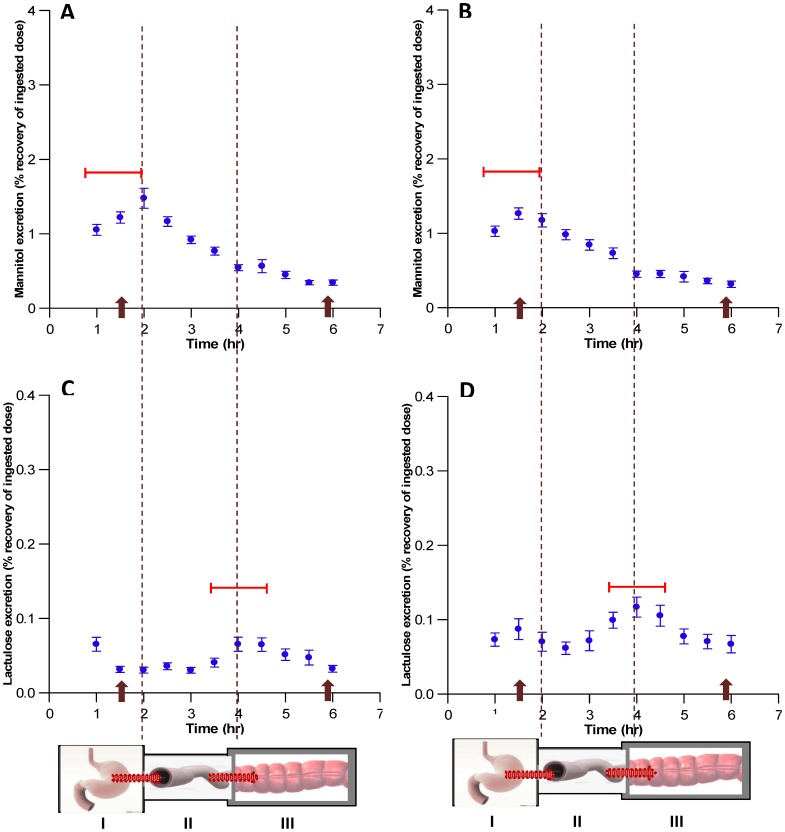
Half-hourly percentage urinary recovery of ingested dose of mannitol (A&B) and lactulose (C&D) in 40 healthy female volunteers following dosage with placebo (A&C) or aspirin (B&D). *The classification of the data into periods (*I, 0–2* *h*, corresponding to the passage of probes from the stomach to the SI; *II, 2½-4* *h,* corresponding to passage from the SI to the colon; *III, 4½-6* *h,* corresponding to the passage from the proximal to distal colon) was based on the early peak in mean % recovery of mannitol and on the later peak in mean % recovery of lactulose viewed in conjunction with the data from SmartPill (see text). Arrows on X axis indicate the time of passage of the SmartPill into subsequent segments of the gut. Dots indicate the mean ±SE of the % half hourly recoveries of the sugars. Horizontal bars indicate the temporal range of the peak in % recovery of each of the sugars between subjects.

The temporal curves of percentage excretion of mannitol and lactulose each exhibited an early and a later peak, the latter being relatively small for mannitol and large for lactulose (Figure 1 A&C). The rise in mannitol excretion, which first peaked at around two hours after dosage, was evident for at least four hours after dosage. It is noteworthy that the position of the initial peak in mannitol excretion varied from 1 to 2 hours post dosage between subjects, both after placebo and aspirin (Figure 1 A&B, represented by the horizontal bars). The initial peak in lactulose excretion was less evident and was overshadowed by a subsequent larger peak which occurred at around 4 h. The position of the larger peak in lactulose excretion varied between subjects from 3½ to 4½ h post dosage both after the consumption of placebo and after the consumption of the aspirin (Figure 1 C&D, represented by the horizontal bars).

Hence the bimodal temporal patterns of the percentage excretion of the two sugars could be categorised into three distinct periods (Figure 1). During the first period, which occurred in the interval between 1 and 2 h after dosage, the percentage recovery of mannitol tended to increase whilst that of lactulose was more variable. During the second period, which occurred in the interval between 2½ and 4 h after dosage, the percentage recovery of mannitol tended to decline whilst that of lactulose tended to increase. During the third period, which occurred in the interval between 4½ and 6 h after dosage, the percentage recoveries of both mannitol and lactulose tended to decrease, the decline in lactulose being more rapid.

### Determination of Retention Time

The mean gastric emptying time of the six subjects determined from the SmartPill data concurrent with dosage with the lactulose mannitol solution was 1.79±0.31 h. This was close to the duration of the first period of the temporal curve and the mean time at which the main peak in mannitol excretion occurred (1.79±0.08 h). Similarly the mean small intestinal emptying time (4.64±0.68 h) was close to that of the duration of the second phase in the temporal curve and the mean time at which the main peak in lactulose excretion occurred (4.30±0.07 h).

### Patterns of Variation of each Sugar within Each Period

**Figure 2:**
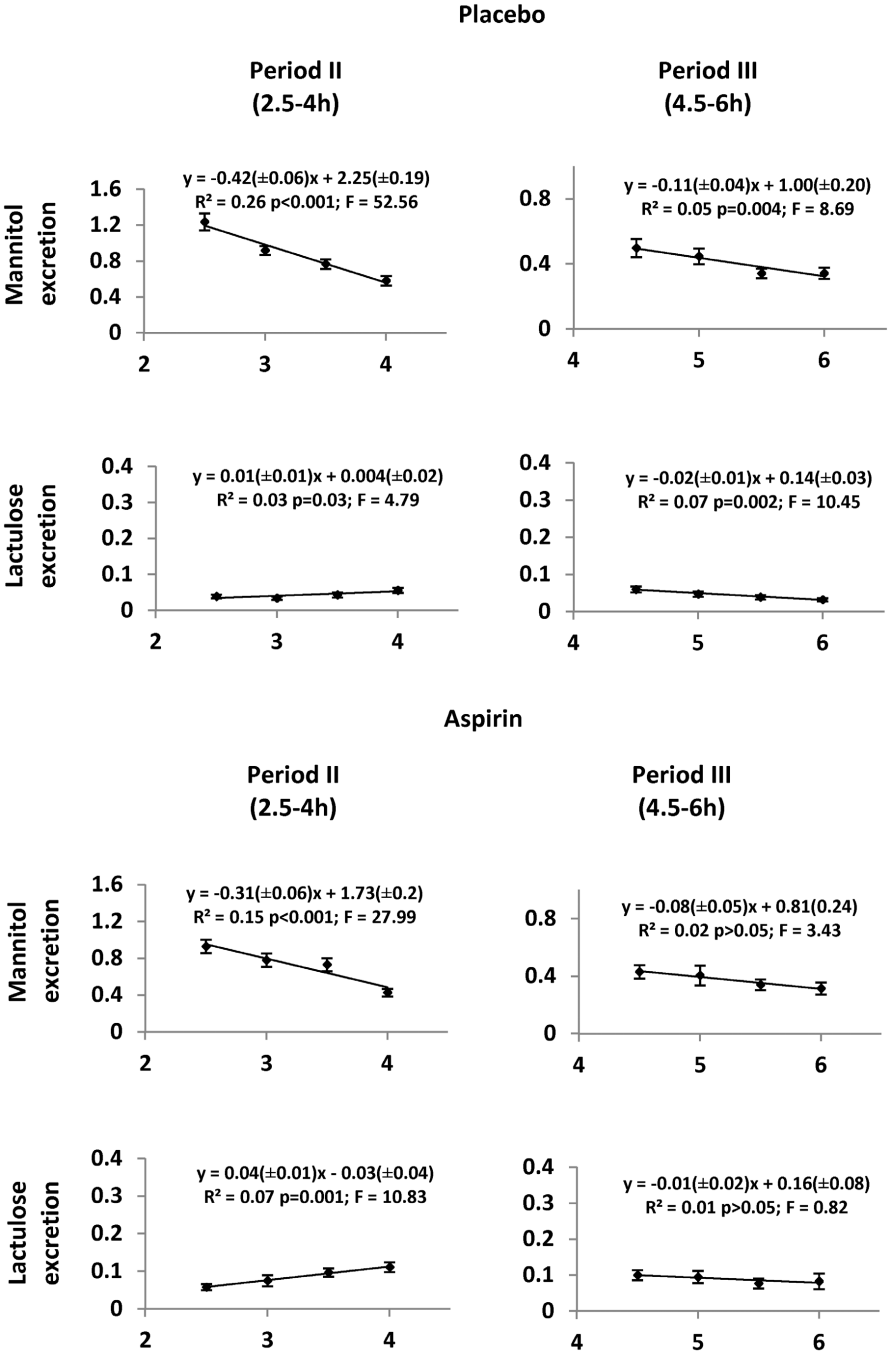
Simple linear regressions of pooled half-hourly percentage urinary recoveries of mannitol and lactulose against time, during period II and period III after dosage with placebo or aspirin, in forty healthy female volunteers. Vertical bars are standard errors.

The temporal profiles of the first period (period I; 0–2 h) did not exhibit consistent patterns between treatments. The temporal profiles of the second (period II; 2½ −4 h) and third (period III; 4½-6 h) periods of the temporal plots of excretion of mannitol were of broadly linear configuration, the percentage excretion tending to decrease with (Figure 1B) and without aspirin (Figure 1A). The excretion rates of mannitol during the period III declined at a significantly lower rate than during period II both with (Figure 2, Table 1) and without aspirin (Figure 2, Table 1). The plots of the excretion rates of lactulose during period II and III differed in configuration from those of mannitol. During period II, lactulose excretion rates showed a tendency to increase, with a greater increase after dosage with aspirin (Figure 2, Table 1). The mean temporal slope of lactulose excretion after the consumption of placebo tended to increase during period II and to decrease during period III (Figure 2). Hence each temporal slope differed significantly from those obtained with mannitol (Period II: d.f 1,18; T  = 7.07; P < 0.001, period III: d.f 1,18; T  = 2.19; P<0.05 respectively). However, after dosage with aspirin, the mean slopes of lactulose excretion differed significantly from those of mannitol only during period II (d.f 1,18; T  = 4.44; P<0.001). It is however noteworthy that the slope of the SLR for the decline in lactulose excretion during period III following consumption of aspirin was not significant (Figure 2). The between subjects standard error was minimised and the correlation coefficient optimised on SLR of values for excretion of both sugars during the 2½ −4 h of collection. This, in conjunction with the finding that the slope of excretion for lactulose differed significantly from that for mannitol during this period, indicates this time period is optimal for urine collection to assess differences in permeability between the two sugars.

**Table 1:** Comparison of results of simple linear regressions from period II and III in the temporal pattern of excretion of lactulose and mannitol in 40 healthy female participants.

Placebo v/s aspirin for each segment					Between periods (II vs. III) for each treatment				
Sugar	Period (h)	t	df	p value	Sugar	Treatment	t	df	p value
Mannitol	II (2.5–4)	1.3	18	NS	Mannitol	Placebo	4.3	18	0.001
	III (4.5–6)	0.47	18	NS		Aspirin	2.95	18	0.009
Lactulose	II (2.5–4)	2.13	18	0.05	Lactulose	Placebo	2.13	18	0.05
	III (4.5–6)	0.45	18	NS		Aspirin	2.23	18	0.04

df = degrees of freedom.

### Variation in Cumulative Recoveries of the Probe Sugars with Duration of Collection

Variations between subjects in the half-hourly percentage recoveries of mannitol, as indicated by the variance and the 95% confidence intervals, were lowest during period II i.e. between the 2½ and 4 h after both aspirin and the placebo. Variation between subjects in half-hourly percentage recoveries of lactulose was similarly lowest during this period.

The cumulative percentage recoveries of mannitol and of lactulose varied with the duration of collection both after placebo and after aspirin. Similarly, the calculated mean hourly percentage recoveries that were derived from these cumulative recoveries varied significantly between subjects at 2 hours and 4 hours after dosage (Table 2). Hence the mean hourly percentage excretion of mannitol declined with time from ingestion both with and without dosage with aspirin (Table 2). Conversely the mean hourly percentage excretion of lactulose increased with time from ingestion after dosage with aspirin but again did not differ after dosage with aspirin from that after dosage with placebo. Correspondingly, these differences caused the overall ratio i.e. LMR to vary with the duration of the period over which urine samples were bulked (Table 2).

**Table 2:** Variation in calculated hourly percentage excretions of urinary lactulose and mannitol with duration of collection period and consequent change in the lactulose:mannitol ratio (LMR) based on data from 40 healthy female participants.

Duration#(hr)	Mean hourly Mannitol		Mean hourly Lactulose	
	Placebo	Aspirin	Placebo	Aspirin
**2**	1.81 (±0.10)	1.97 (±0.14) *	0.10 (±0.01)^ †^	0.15 (±0.02)* ^†^
**4**	1.73 (±0.08)	1.68 (±0.10) *	0.09 (±0.01)^ †^	0.16 (±0.02)* ^†^
	**LMR**			
	**Placebo**	**Aspirin**		
**2**				
Ratio Range	0.053–0.058	0.071–0.081*		
**4**				
Ratio Range	0.049–0.054	0.091–0.099*		

Data are the mean ±SE.

#Duration: 2 h = (1/2 h–2 h)/2; 4 h = (1/2 h–4 h)/4.

*P<0.001∶2 h versus 4 h; ^†^P<0.001: placebo versus aspirin.

### Comparisons with Published Values

**Figure 3:**
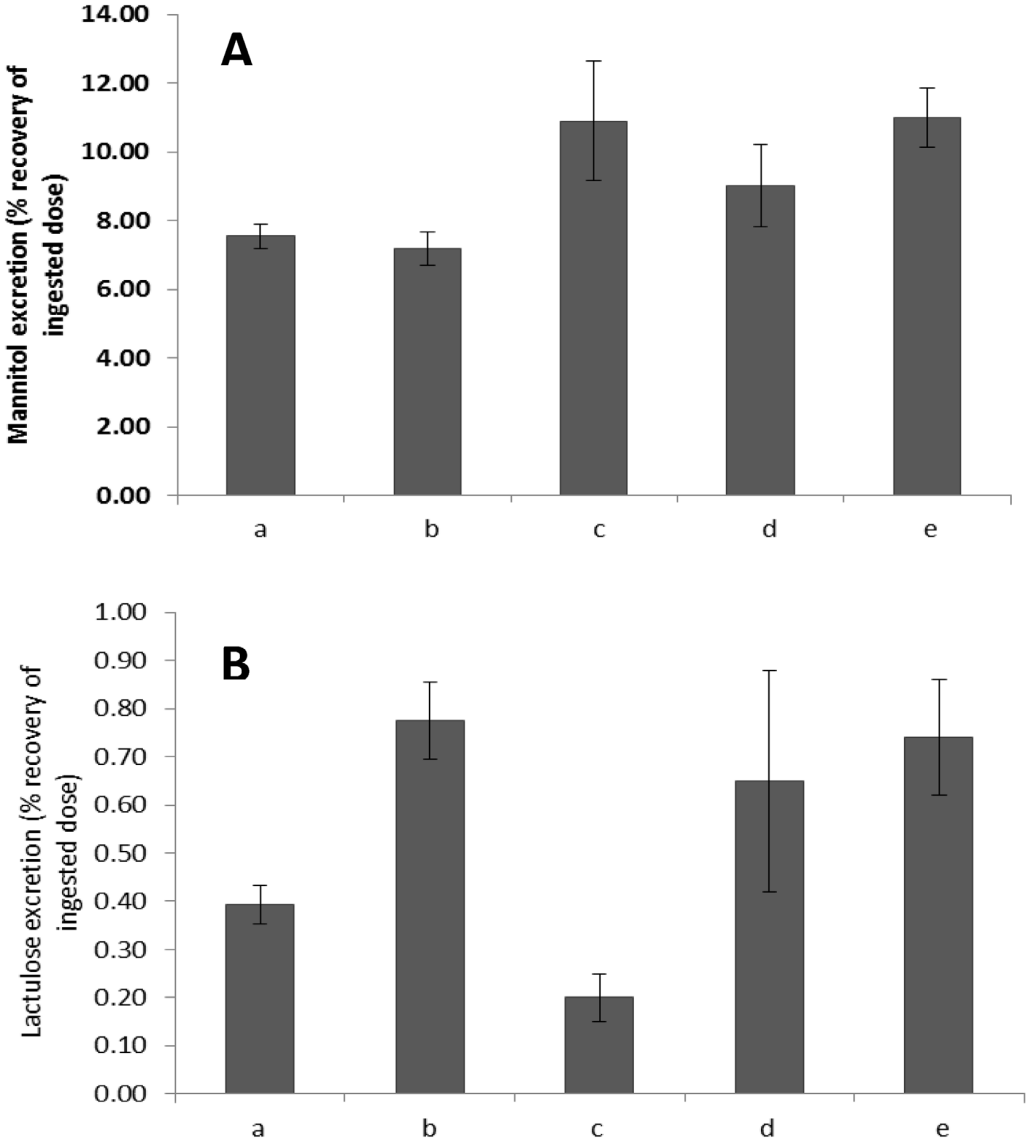
Comparison of calculated 5 h cumulative recoveries of mannitol (A) and lactulose (B) in forty healthy female volunteers following dosage with placebo (a) or 600 mg aspirin (b) with those reported for subjects with pro-inflammatory conditions i.e. coeliac disease (c [40], d [42]) and Crohn’s disease(e [41]).

The augmented cumulative 5 h lactulose excretions that were obtained in our studies after dosage with aspirin, were similar to those reported in patients with Crohn’s disease [41] (Figure 3) but significantly higher (d.f 2,52; T  = 4.17; P < 0.001) than those in the study of subjects with coeliac disease [40]. However the cumulative 5 h mannitol excretions in the study of the subjects with Crohn’s disease [41] were significantly (d.f 2,85; T  = 3.17; P < 0.001) higher than those of our healthy subjects without aspirin. The reported mean LMR from the study of subjects with Crohn’s disease (0.067) [41] was lower than that from subjects in our study who had received aspirin (0.108). Again the cumulative 5 h mannitol excretion in the study of subjects with coeliac disease [40] was significantly higher than those of the subjects in our study both in the control (d.f 2,52; T  = 2.84; P = 0.006) and aspirin (d.f 2,52; T  = 2.85; P = 0.006) treatments in our study.

**Figure 4:**
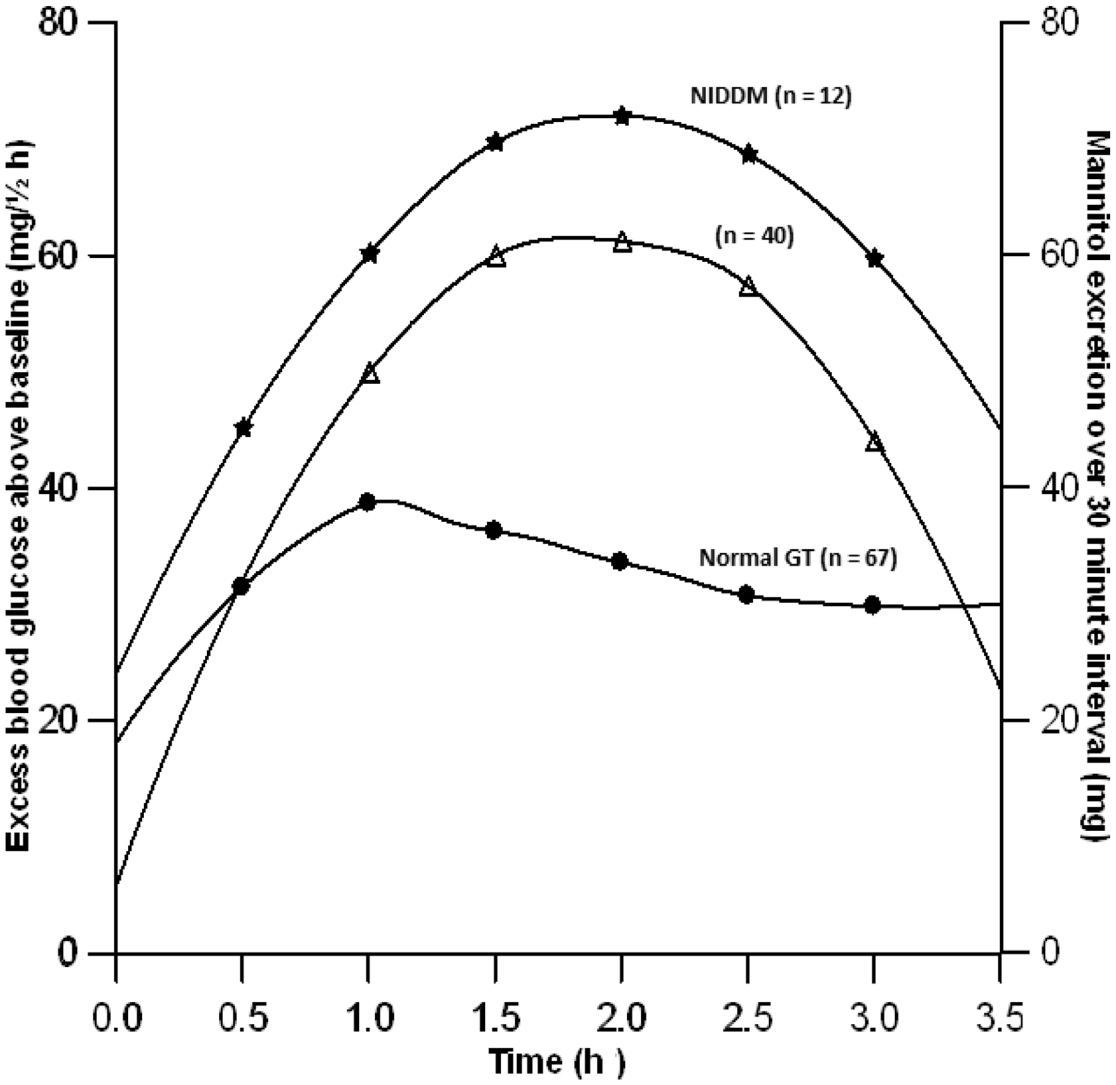
Comparison of temporal patterns of half-hourly urinary mannitol (mg) excretion from 40 healthy female volunteers with calculated half-hourly glucose absorbed (mg/½h) based on published blood sugar values of women with non-insulin dependent diabetes (NIDDN, n = 12) [44] and those with normal glucose tolerance (NGT; n = 67) [44].

Graphic comparison of integrated half-hourly glucose levels from fifteen subjects with non-insulin dependent diabetes mellitus [44] with those of half-hourly mannitol excretion obtained from our study, showed that both peaked at approximately two hours post dosage as would be expected if active absorption of glucose was inhibited and glucose absorbed passively (Figure 4). Conversely graphic comparison of the integrated half-hourly glucose levels of sixty seven healthy women (n = 67) [44] with those of half hourly mannitol excretion from this study showed that glucose levels peaked earlier than did those of mannitol and were subsequently attenuated, this presumably resulting from active glucose uptake and disposal (Figure 4).

## Discussion

The results of this study, notably the minimisation of the between subject standard error, the optimisation of the between subject correlation between quantities of each sugar excreted with time and the significant between subject difference in the temporal patterns of excretion of the two sugars during this time period, indicate that the optimal period for collection of urine used in clinical tests of small intestinal permeability is between 2½–4 h post dosage. The variation in the LMR would be similarly minimised. Moreover, in the light of the concurrent SmartPill studies, the characteristics of the temporal profiles of the excretion of the two sugars suggest that this period is optimal as it avoids incorporation of gastric residence time which varies greatly between individuals. Hence, the commencement of collection at 2½ will ensure that in the majority of subjects the bulk of the dose of each probe will have exited the stomach and the column of digesta containing them will be advancing steadily through the small and large intestine, the low inter-subject variation reflecting the relative lack of variability between subjects in the rates of passive absorption from these structures.

Given that the permeability of the small intestine (SI) to mannitol is higher than that of the large intestine (LI) [22] and that the permeability of the LI to lactulose is higher than that of the SI [22], the temporal profiles of the half-hourly percentage excretion of the two saccharidic probes, fit with a hypotheses that a column of digesta occupies an increasing length of small intestine as it leaves the stomach [13], [45] and that the leading edge of this column is transiting from the small to the larger bowel at the time when the trailing edge has left the stomach.

This conclusion, that a column of digesta containing the probe occupies and is absorbed from, a length of intestine, is supported by a body of data describing the quantitative dependence of hormonal feedback loops that modulate gastric emptying on the length of intestine that is occupied by nutrient containing chyme. Hence the rate of gastric emptying is dependent upon the rate of absorption of nutrients [46] such as glucose [47] or fat [48] from the volume of digestate [49] that is in contact with small intestinal mucosa and thus to the length of small intestinal lumen that contains it.

The fact that the mean retention time of the SmartPill (1.79 h) was close to the mean time when the absorption of mannitol was maximal (2 hours after dosage) also fits with this hypothesis given that the SmartPill is likely have been expelled from the stomach after the bulk of the fluid phase was expelled [37], [38] . Hence, as stated hitherto, the collection period that minimises variance between subjects will occur when, in most subjects, the column of digesta containing the probes is transiting from the small to the large intestine. Again the rate at which the excretion of mannitol decreases and that of lactulose increases during this period will depend on the rate at which digesta transits from the small to the large intestine which appears to be relatively invariant between subjects and not to be influenced by aspirin.

We could find no published data for gastric emptying time after the consumption of 100 ml of clear fluid containing simple sugars. However mean gastric emptying time evaluated by the [^13^C] acetate breath test following a 250 ml liquid meal (Fresubin diabetes, Fresenius, Oberursel, Germany) containing protein, carbohydrate and fat (225kcal) is reported to be 1.28±0.31 h [50]. Given that the volume of the gastric load in relation to the volume of the stomach [51], [52] and the nutrient content of the meal would influence gastric emptying [53]–[55], this value is broadly compatible to that obtained in our study.

The calculated five hour cumulative excretions of the two sugars based on our data are broadly similar to those reported in subjects with pathological disturbances in permeability [41]. However the importance of examining lactulose and mannitol excretion separately [22] as well as in simple ratio is manifest in these comparisons. Hence the cumulative 5 h lactulose excretion reported in subjects with Crohn’s disease [41] was similar to that in our study following dosage with aspirin whilst the cumulative 5 h excretion of mannitol was higher than that in our healthy subjects. This caused the mean LMR of 0.067 [41] of subjects with Crohn’s disease to be lower than that in our study after aspirin (0.108) and runs contrary to the hypotheses that the absorptive surface area is compromised by inflammatory disease [16]–[18]. However, it is noteworthy that this study included patients with differing Harvey Bradshaw grades of Crohn’s disease and included those in remission as well as those with mildly active disease. Again the sites of the lesions varied, some participants having both small and large bowel lesions [41]. Further, the reported values are from studies that included both male and female patients whereas our study included only females.

The fact that the 5 h cumulative lactulose excretion reported in one study of subjects with frank coeliac disease was lower than those in our studies and their mannitol excretions higher giving LMRs of 0.146 [42] and 0.018 [40] is further testament to the variability of the LMR and the variability of the excretion of component sugars with differing inflammatory stimuli. These examples likely indicate that inflammation from a disease and from a pro-inflammatory stimulus such as aspirin can have differing outcomes that may be better identified by comparisons based on the temporal profiles of the two probes rather than the ratios of cumulative excretion. Hence for example, inflammatory disorders of the intestine may have greater influence on the rate of transit of the column of digesta containing the probes through particular segments of the gut. In regard to the latter and in regard to the variability in gastric emptying there is a need for further research, for example to determine whether there is a place for simultaneous use of the SmartPill in determinations of permeability.

The absorption of mannitol is passive [9], [56] and dependent largely on the concentration of the sugar in the periphery of the lumen. As such it can provide an index of the extent of mixing the column of digesta within the lumen that is useful in assessing effects from the physical form of the food or pharmaceutical formulation on absorption. Unlike that of glucose, the temporal profile of mannitol absorption is not augmented by hormonal effects such as those from insulin/incretin [57], [58] on active transporters. This is readily illustrated by comparisons of the half-hourly percentage cumulative excretion profile of mannitol with published blood sugar values from oral glucose tolerance tests [44] that have been integrated [43] to convert them from instantaneous rate of absorption/storage to the cumulative quantity of glucose required to maintain the half-hourly profile above the baseline value. Graphic comparison of integrated half-hourly glucose levels from twelve subjects with non-insulin dependent diabetes mellitus [44] with those of half-hourly mannitol excretion obtained from our study shows that both peaked at approximately two hours post dosage as would be expected if the active absorption or disposal of glucose was inhibited. Conversely graphic comparison of the integrated half-hourly glucose levels of sixty seven healthy women [44] with those of half-hourly mannitol excretion from this study showed the glucose levels of healthy subjects peaked earlier and were subsequently attenuated, this presumably resulting from normal incretin and insulin mediated augmentation of glucose uptake and disposal.

In summary the results of this study indicate that in healthy subjects the quantities of lactulose and mannitol absorbed from the intestine and excreted in the urine, and the ratios between them, may be confounded by differences in the time periods over which they are collected. Hence collection periods should be standardized for use in clinical tests. In this regard the period of highest levels of consistency of absorption between subjects in assays of the absorption and urinary excretion of lactulose and mannitol was between 2.5 and 4 h judged on the basis of minimal standard error and maximal R^2^ values of SLR. This period, was shown by concurrent dosage with the SmartPill to correspond to the period when the column of digesta containing the probes was passing from the small to the large intestine. Further, that comparison of the two sugars should include consideration of the absolute amounts of each sugar that are absorbed rather than be restricted to the ratio of the two, as the amounts of each may be influenced differently by disease. Finally, that comparisons of the temporal patterns of sugars that are passively absorbed such as mannitol with those that are actively absorbed such as glucose may be useful in understanding the effects of diseases such as diabetes mellitus on the mucosal dynamics of absorption.
